# Patterm of occurrence of occupational exposure to blood-borne pathogens in a tertiary hospital in Saudi Arabia

**DOI:** 10.1186/1753-6561-5-S6-P218

**Published:** 2011-06-29

**Authors:** WA Mazi, AT Abatayo, N Kazem, A Senok

**Affiliations:** 1 Infection Prevention and Control Department, King Abdul Aziz Specilaist Hospital, Saudi Arabia; 2 Staff Clinic Department, King Abdul Aziz Specialsit Hospital, Taif, Saudi Arabia; 3 College of Medicine, Al Faisal University, Riyadh, Saudi Arabia

## Introduction / objectives

Occupational exposure to blood borne pathogens (OEBBPs) remains a common occurrence globally despite the introduction of safety needle devices. We assessed the causative factors and pattern of occurrence of OEBBPs at King Abdul Aziz Specialist Hospital (KAASH), Taif Saudi Arabia.

## Methods

We assessed the occurrence of OEBBPs at KAASH from January 2009 to December 2010. During this period, data on reported cases was collected using the Center for Disease Control and Prevention (CDC) OEBBPS notification form.

## Results

106 episodes of OEBBPs were reported comprising of 41 cases in 2009 and 65 in 2010. The incidence of OEBBPs was 4.09/10000 patient-days and 5.90/10000 patient-days in 2009 and 2010 respectively. This increased incidence in 2010 exceeded our internal of OEBBPs incidence benchmark of 3.00/10000 patient-days. Majority of episodes were recorded among the nursing staff (82%). The highest number of episodes were from the emergency room (21.5%) followed by the kidney center (16.9%). Majority of episodes were reported during the morning shift, occurring during specimen collection by venipuncture and while establishing intravenous or arterial access. Post exposure prophylaxes against hepatitis B virus have been given immediately as indicated. laboratory tests for diagnosis hepatitis C virus seroconversion have been done. No case exposure to HIV positive was recorded. No case of seroconversion has been documented.

## Conclusion

Introduction vaccutainer blood collection method and needless devices as well as an OEBBPs notification hotline are recommended to address this high level of OEBBPs. In early 2011, a sharp injuries prevention campaign was conducted and evaluation of its efficacy in reducing the incidence of OEBBPs in our hospital is ongoing.

## Disclosure of interest

None declared.

